# A Nomogram-Based Risk Classification System Predicting the Overall Survival of Patients With Newly Diagnosed Stage IVB Cervix Uteri Carcinoma

**DOI:** 10.3389/fmed.2021.693567

**Published:** 2021-07-15

**Authors:** Wenke Yu, Lu Huang, Zixing Zhong, Tao Song, Hong'en Xu, Yongshi Jia, Jinming Hu, Huafeng Shou

**Affiliations:** ^1^Department of Radiology, Zhejiang Qingchun Hospital, Hangzhou, China; ^2^Department of Gynecology, Zhejiang Provincial People's Hospital, Affiliated People's Hospital, Hangzhou Medical College, Hangzhou, China; ^3^Department of Obstetrics, Zhejiang Provincial People's Hospital, Affiliated People's Hospital, Hangzhou Medical College, Hangzhou, China; ^4^Department of Radiation Oncology, Oncology Center of Zhejiang Provincial People's Hospital, Affiliated People's Hospital, Hangzhou Medical College, Hangzhou, China

**Keywords:** survival, risk model, cancer risk factors, nomogram, SEER

## Abstract

**Background:** This study constructed and demonstrated a model to predict the overall survival (OS) of newly diagnosed distant metastatic cervical cancer (mCC) patients.

**Methods:** The SEER (Surveillance, Epidemiology, and End Results) database was used to collect the eligible data, which from 2010 to 2016. Then these data were separated into training and validation cohorts (7:3) randomly. Cox regression analyses was used to identify parameters significantly correlated with OS. Harrell's Concordance index (C-index), calibration curves, and decision curve analysis (DCA) were further applied to verify the performance of this model.

**Results:** A total of 2,091 eligible patients were enrolled and randomly split into training (*n* = 1,467) and validation (*n* = 624) cohorts. Multivariate analyses revealed that age, histology, T stage, tumor size, metastatic sites, local surgery, chemotherapy, and radiotherapy were independent prognostic parameters and were then used to build a nomogram for predicting 1 and 2-year OS. The C-index of training group and validation group was 0.714 and 0.707, respectively. The calibration curve demonstrated that the actual observation was in good agreement with the predicted results concluded by the nomogram model. Its clinical usefulness was further revealed by the DCAs. Based on the scores from the nomogram, a corresponding risk classification system was constructed. In the overall population, the median OS time was 23.0 months (95% confidence interval [CI], 20.5–25.5), 12.0 months (95% CI, 11.1–12.9), and 5.0 months (95% CI, 4.4–5.6), in the low-risk group, intermediate-risk group, and high-risk group, respectively.

**Conclusion:** A novel nomogram and a risk classification system were established in this study, which purposed to predict the OS time with mCC patients. These tools could be applied to prognostic analysis and should be validated in future studies.

## Introduction

Cervix uteri carcinoma is the fourth most common gynecological cancer in the world, with an estimated 569,847 new cases and 311,365 deaths in 2018 (1, 2). Although the human papillomavirus (HPV) vaccine and cervical cytological screening have decreased the morbidity of cervical uterine carcinoma, distant metastatic cervical carcinoma (mCC, stage IVB) remains a major cause of cancer-related death among women globally.

How best to manage mCC is currently under debate due to its notorious biological behavior and highly heterogeneous clinical manifestations (3). Although the Gynecologic Oncology Group 204 trial had shown that the bevacizumab combination with chemotherapy (CT) could prolong the OS over 12 months for metastatic, persistent, or recurrent cervical uterine carcinoma (4), the prognosis of mCC patients remains poor and most patients still adopt palliative treatment at present. Additionally, the high cost of adding bevacizumab to the treatment has prohibited its wide application in developing areas (5). On the other hand, for mCC patients with only invaded distant lymph nodes, a small sample retrospective study showed that double therapy consisting of radiotherapy (RT) and CT could achieve a significant long-term survival benefit over CT alone (63.7 months median OS vs. 18.4 months, respectively) (6). For mCC patients diagnosed with hematogenous dissemination to the pulmonary system after curative initial treatment, a previous study demonstrated that the 5-year disease-free survival rate after pulmonary metastasectomy was 32.9% (7). Combined with medical progress in better management of other metastatic lesions (8, 9), individualized treatment strategies are meaningful and urgent for mCC.

Currently, statistical improvements, such as nomograms, have been widely developed and applied in oncological practice for prognostic prediction of a specific clinical endpoint (10). Moreover, the Surveillance, Epidemiology and End Results (SEER) database includes data on cancer diagnosis, patient demographics (age, race, insurance, etc.), tumor characteristics (location, grade, tumor stage, metastatic sites, etc.), treatment strategies (use of surgery, RT, CT) and survival records for nearly 30% of the U.S. population; thus, this database has unique advantages in cancer research (11, 12). For patients with mCC registered in the SEER database, a previous study found that several parameters were significantly correlated with OS (such as age, pathological type, metastatic numbers, RT, CT, etc.) (13). However, debates about the value of local surgery and the metastatic sites involved still need to be addressed with more clinical data. Based on this background, we extracted data from the SEER database to explore potential risk parameters that are significantly correlated with mCC and further constructed and demonstrated a prognostic model to predict the OS time of patients with mCC.

## Methods

### Ethics Statement

The Institutional Review Board of Zhejiang Provincial People's Hospital exempted this study from informed consent given that data in the SEER database (SEER^*^Stat version 8.3.6) are de-identified and publicly available after receiving permission for their use (Reference number: 10579-Nov2019). We confirm that this study was performed in accordance with the Declaration of Helsinki.

### Study Population

Eligible data was obtained according to the following criteria: (1) pathological diagnosis of cervical uterine carcinoma by morphological code C53.9 between 2010 and 2016; (2) a primary diagnosis of cervical uterine carcinoma; and (3) IVB diseases diagnosed according to the 7th edition criteria of the TNM classification of malignant tumors and SEER Combined Stage (2016+). The major exclusion criteria were defined as follows: (1) mCC patients who were diagnosed with more than one primary cancer and for whom cervical uterine carcinoma was not their first diagnosed cancer; or (2) incomplete data registered in the database. Flow chart of the process of data extraction from the SEER database is presented in Supplementary Figure 1. The year 2010 was chosen as the first year given that recodes for metastatic sites were collected from the items of the “SEER Combined Metastasis at Diagnosis (2010+)” for bone, brain, liver and lung.

Additionally, for mCC patients diagnosed between 2010 and 2015, the variables of “metastatic sites” and “tumor size” were recorded as “CS metastasis at diagnosis (2004–2015)” and “CS tumor size (2004–2015)” based on the Collaborative Stage Data Collection System, which is available online (version 02.05). In particular, we reclassified the status of the metastatic sites into four groups: “Only lymph nodes (LN),” “Only organ,” “Both,” and “Unknown.” Similarly, for mCC patients diagnosed in 2016, the variable “metastatic sites” was reclassified based on the criteria of “Metastasis at diagnosis-Distant LN (2016+)” and “Metastasis at diagnosis-Other (2016+).” The variables “Age at diagnosis,” “Insurance status,” “Race,” “Marital status,” “Tumor histology,” “Differentiation,” “T stage,” “N stage,” “Surgery at the primary site,” “delivery of RT,” and “use of CT” were as described previously (14, 15). Following our previous study, we set 65 years old as the cutoff point for the age at diagnosis in the current study. Additionally, median tumor size was applied to divide patients into two different groups, except for patients with unknown data.

### Statistical Analysis

The primary endpoint was set to OS. It was calculated as the duration from the diagnosis of cervical uterine carcinoma to death or the last follow-up registered in the SEER database. After enrollment, eligible data were randomly divided into training and validation cohorts according to the ratio of 7:3. Then, data in the training cohort was used to train and construct the predictive model and build the nomogram and risk classification system based on the results obtained in the multivariate analyses. The data of validation group was used to further verify the prediction model.

The predictive model was built as follows: first, a univariate analysis was conducted to obtain the predictive parameters for predicting OS. Parameters identified with significant difference (*P* ≤ 0.05) in the univariate analysis were then integrated into the multivariate Cox regression analyses with a backward model selection procedure. Factors with a *p* ≤ 0.05 were considered significant to obtain the multivariate Cox regression model. Finally, these independent predictors were integrated to build the nomogram model to predict the 1 and 2-year OS.

Secondly, the risk classification system was generated based on the total scores of each patient by using the nomogram to stratify mCC patients into three different risk groups (low-risk group, intermediate-risk group, and high-risk group). The cutoff values were identified with the X-tile program (developed by Yale University, version 3.6.1) and further validated in the validation cohort. This program has been demonstrated to be a convenient and reliable tool for biomarker assessment and outcome-based cut point optimization (16). Kaplan-Meier method was used to calculate the survival estimation among different predictive factors and compared by the log-rank test.

The validation of the nomogram model was evaluated using the Harrell's concordance index (C-index), calibration curves, and decision curve analyses (DCAs). C-index values ≥0.7 indicate a comparatively accurate prediction in general (17, 18). Calibration curves (bootstrap analyses with 1,000 resamples) were integrated to test the calibration of the nomogram model, and DCAs were further used to evaluate the usefulness of the model constructed by previous steps.

All statistical analyses were performed using R software version 3.6.2 (https://www.r-project.org) and the IBM SPSS statistical software package, version 25.0 (SPSS, Armonk, New York, USA). The survival curves were compared and drawn with GraphPad Prism 8.0 (GraphPad Software, San Diego, CA, USA), and a two-sided *p* < 0.05 was considered significant.

## Results

### Baseline Characteristics

A total of 2,091 mCC patients registered in the SEER database were identified and enrolled in this analysis between 2010 and 2016. The clinicopathologic characteristics of the mCC patients in the training and validation cohorts were demonstrated in Table 1. No significant differences were observed between these two groups (*P* > 0.05).

**Table 1:** Baseline characteristics of the mCC patients.

**Characteristic**	**Frequency (*n*, %)**	**Training cohort (*n*, %)**	**Validation cohort (*n*, %)**	***P***
Age (years)				0.213
Median (IQR)	55 (45–64)			
<65	1,581 (75.6)	1,098 (74.8)	483 (77.4)	
≥65	510 (24.4)	369 (25.2)	141 (22.6)	
Insurance				0.726
Insured	1,117 (53.4)	780 (53.2)	337 (54.0)	
Uninsured and others	974 (46.6)	687 (46.8)	287 (46.0)	
Marital status				0.866
Married	716 (34.2)	504 (34.4)	212 (34.0)	
Unmarried and others	1,375 (65.8)	963 (65.6)	412 (66.0)	
Race				0.370
White	1,506 (72.0)	1,065 (72.6)	441 (70.7)	
Non-white	585 (28.0)	402 (27.4)	183 (29.3)	
Histology				0.957
SCC	1,466 (70.1)	1,028 (70.1)	438 (70.2)	
Non-SCC	625 (29.9)	439 (29.9)	186 (29.8)	
Differentiation				0.628
Well and fairly	490 (23.4)	336 (22.9)	154 (24.7)	
Poorly and undifferentiated	938 (44.9)	659 (44.9)	279 (44.7)	
Unknown	663 (31.7)	472 (32.2)	191 (30.6)	
T stage				0.927
T1-2	720 (34.4)	509 (34.7)	211 (33.8)	
T3-4	1,092 (52.2)	763 (52.0)	329 (52.7)	
Tx	279 (13.4)	195 (13.3)	84 (13.5)	
N stage				0.583
Negative	513 (24.5)	353 (24.1)	160 (25.6)	
Positive	1,352 (64.7)	950 (64.8)	402 (64.4)	
Nx	226 (10.8)	164 (11.1)	62 (10.0)	
Surgery at the primary site				0.354
Local surgery	294 (14.1)	213 (14.5)	81 (13.0)	
No/Unknown	1,797 (85.9)	1254 (85.5)	543 (87.0)	
Radiotherapy (RT)				0.792
Yes	1,392 (66.6)	974 (66.4)	418 (67.0)	
No/unknown	699 (33.4)	493 (33.6)	206 (33.0)	
Chemotherapy (CT)				0.257
Yes	1,581 (75.6)	1,099 (74.9)	482 (77.2)	
No/unknown	510 (24.4)	368 (25.1)	142 (22.8)	
Tumor size (mm)^*^				0.083
<63	614 (29.4)	426 (29.0)	188 (30.1)	
≥63	607 (29.0)	409 (27.9)	198 (31.7)	
Unknown	870 (41.6)	632 (43.1)	238 (38.2)	
Metastatis				0.879
Only LN	701 (33.5)	489 (33.3)	212 (34.0)	
Only organ	605 (28.9)	432 (29.5)	173 (27.7)	
Both	525 (25.1)	364 (24.8)	161 (25.8)	
Unknown	260 (12.5)	182 (12.4)	78 (12.5)	

*IQR, interquartile range; SCC, squamous cell carcinoma; LN, lymph node*.

* *Median tumor size is 63 mm*.

### Nomogram Development and Validation

**Figure 1:**
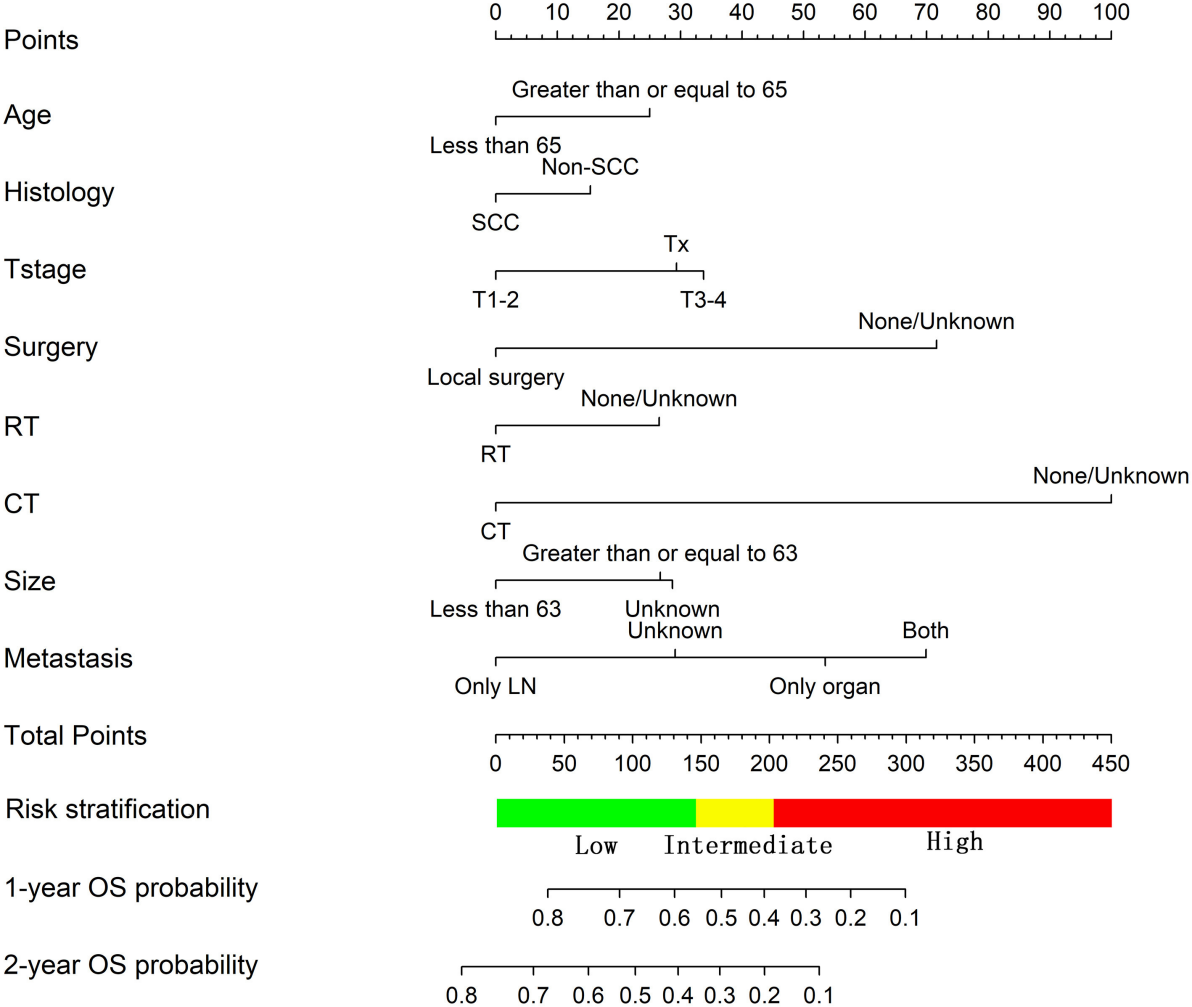
Nomogram predicting the OS of patients with newly diagnosed, distant metastatic cervical carcinoma in the training group.

**Figure 2:**
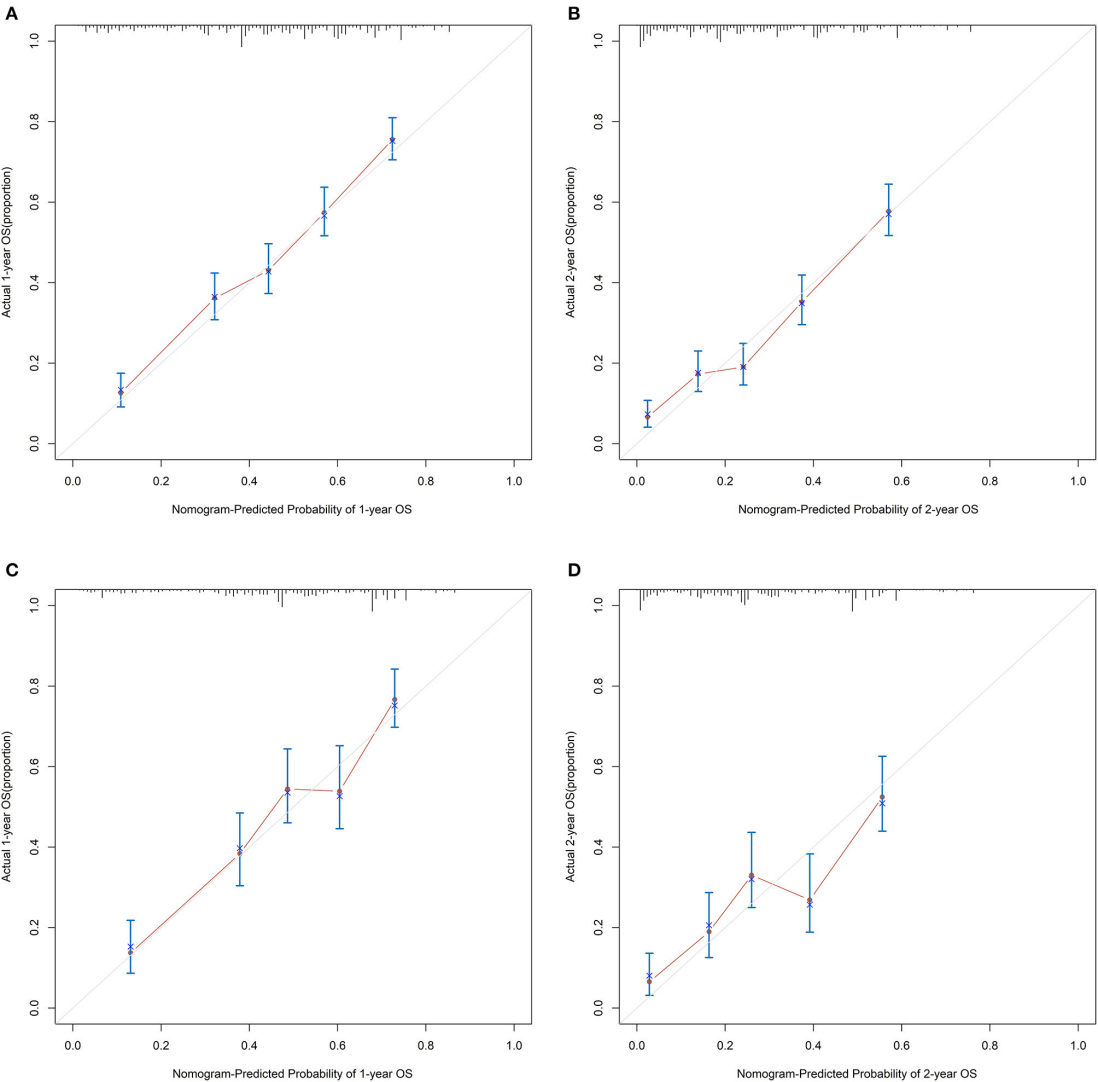
The calibration curves for predicting patients' OS at 1- and 2-year in the **(A,B)** training group and **(C,D)** validation group, respectively.

**Figure 3:**
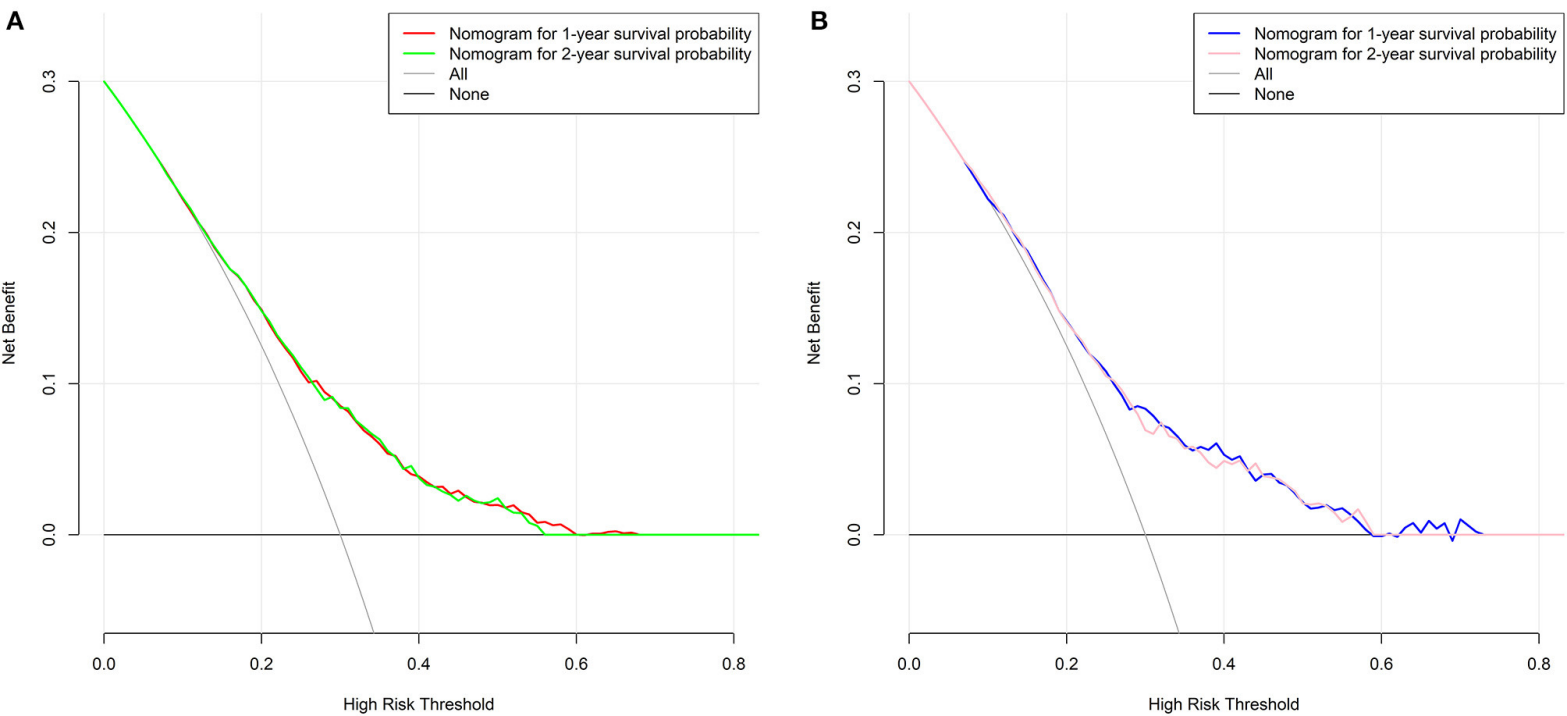
Decision curves of the nomogram predicting 1 and 2-year OS in the **(A)** training group and **(B)** validation group, respectively.

For the training cohort, univariate analyses showed that baseline characteristics including age at diagnosis (*P* < 0.001), marital status (*P* < 0.001), tumor histology (*P* = 0.003), T stage (*P* < 0.001), N stage (*P* < 0.001), tumor size (*P* < 0.001), metastatic sites (*P* < 0.001), and treatment-related factors like surgery at the primary site (*P* < 0.001), RT (*P* < 0.001), and CT (*P* < 0.001) were significantly correlated with OS (Table 2). In the multivariate Cox regression analysis, the prognostic parameters significantly related to the OS were: age at diagnosis (*P* = 0.006, HR = 1.220), tumor histology (*P* = 0.005, HR = 1.147), T stage (T1-2 vs. T3-4, *P* < 0.001, HR= 1.348; T1-2 vs. Tx, *P* = 0.020, HR= 1.043), surgery at the primary site (*P* < 0.001, HR = 1.832), RT (*P* = 0.002, HR= 1.246), CT (*P* < 0.001, HR = 2.342), tumor size (<63 vs. ≥63, *P* = 0.012, HR= 1.248; <63 vs. unknown, *P* = 0.003, HR= 1.271), and metastatic sites (only LN vs. only organ, *P* < 0.001, HR = 1.642; only LN vs. both, *P* < 0.001, HR = 1.827; only LN vs. unknown, *P* = 0.019, HR = 1.298). Then, a nomogram for predicting 1 and 2-year OS that integrated all eight independent prognostic parameters as revealed by the Cox regression was built. CT (nomogram score range from 0 to 100.0) was shown to be the most important prognostic parameter for OS estimation, followed by surgery at the primary site (0–71.605), metastatic sites (0–69.882), T stage (0–33.738), tumor size (0–28.679), RT (0–26.509), age at diagnosis (0–24.985) and tumor histology (0–15.362; Figure 1). The C-index in the training and validation cohorts was 0.714 and 0.707, respectively. Calibration plots showed satisfactory agreement between the prediction and actual survival in this population (Figure 2). The DCAs exhibited that this prediction model has gained great benefits for predicting 1 and 2-year OS time within all of the threshold probabilities and added more positive net benefit than the “all” or “none” strategies between the training and validation groups (Figure 3).

**Table 2:** Univariate and multivariate analyses of OS in the training group.

	**Overall survival (OS)**							
	**Univariate**				**Multivariate**			
	***P***	**HR**	**95% CI**	**95% CI**	***P***	**HR**	**95% CI**	**95% CI**
**Factor**			**Lower**	**Upper**			**Lower**	**Upper**
Age at diagnosis, <65 vs. ≥65	<0.001	1.448	1.262	1.662	0.006	1.220	1.057	1.407
Insurance, insured vs. uninsured and others	0.237	1.076	0.953	1.216	-			
Marital status, married vs. unmarried and others	<0.001	1.264	1.110	1.439	0.096	1.120	0.980	1.279
Race, white vs. non-white	0.185	1.096	0.957	1.256	-			
Histology, SCC vs. Non-SCC	0.003	1.218	1.068	1.390	0.050	1.147	1.000	1.313
Differentiation, reference: well and fairly	0.108				-			
Poorly and undifferentiated	0.135	1.127	0.963	1.319				
Unknown	0.036	1.197	1.012	1.416				
T stage, reference: T1-2	<0.001				<0.001			
T3-4	<0.001	1.582	1.377	1.816	<0.001	1.348	1.166	1.558
Tx	<0.001	2.085	1.713	2.537	0.020	1.312	1.043	1.650
N stage, reference: negative	<0.001				0.162			
Positive	0.886	1.011	0.873	1.170	0.058	1.160	0.995	1.352
Nx	<0.001	1.568	1.264	1.945	0.351	1.119	0.884	1.416
Surgery at the primary site, local surgery vs. no/unknown	<0.001	2.143	1.759	2.612	<0.001	1.832	1.494	2.245
Radiotherapy, yes vs. no/unknown	<0.001	1.624	1.430	1.846	0.002	1.246	1.084	1.433
Chemotherapy, yes vs. no/unknown	<0.001	2.653	2.319	3.035	<0.001	2.342	2.025	2.709
Tumor size (mm), reference: <63	<0.001				0.008			
≥63	<0.001	1.394	1.179	1.648	0.012	1.248	1.051	1.482
Unknown	<0.001	1.631	1.402	1.897	0.003	1.271	1.083	1.492
Metastatic sites, reference: only LNs	<0.001				<0.001			
Only organ	<0.001	1.967	1.678	2.306	<0.001	1.642	1.386	1.946
Both	<0.001	2.153	1.825	2.540	<0.001	1.827	1.540	2.166
Unknown	<0.001	1.817	1.472	2.242	0.019	1.298	1.044	1.614

*SCC, squamous cell carcinoma; LNs: lymph nodes; HR, hazard ratio; CI, confidence interval*.

### Risk Classification System

**Figure 4:**
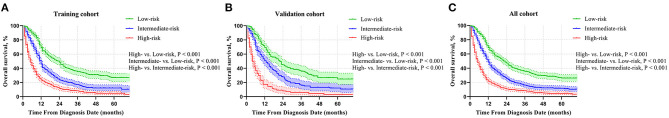
Comparison of OS in the low-risk, intermediate-risk, and high-risk group in the **(A)** training group, **(B)** validation group, and **(C)** all patients.

We further calculated the risk scores based on the nomogram model for each mCC patient to construct a risk classification system and divided enrolled patients into three risk groups: low-risk, intermediate-risk, and high-risk, respectively. According to the cutoff analyses for the training group by the X-title program (Supplementary Figure 2), the cutoff points were classified as follows: <145 (<145), between 145 and 203 (145 ≤ nomogram score <203), and ≥203 (≥203). In the training group, the median OS time of the mCC patients in the low-risk, intermediate-risk, and high-risk group was 24.0 months (95% CI, 20.4–27.6), 12.0 months (95% CI, 11.1–12.9), and 5.0 months (95% CI, 4.3–5.7), respectively, and the 1-year OS rate in these three risk groups was 69.2% (95% CI, 0.647–0.737), 46.1% (95% CI, 0.412–0.510), and 23.4% (95% CI, 0.197–0.271), respectively (Figure 4A). In the validation group, the median OS time of the mCC patients in the low-, intermediate-, and high-risk groups was 22.0 months (95% CI, 17.4–26.6), 13.0 months (95% CI, 10.6–15.4), and 4.0 months (95% CI, 3.1–4.9), respectively, and the 1-year OS rate in the three risk groups was 69.4% (95% CI, 0.629–0.759), 50.6% (95% CI, 0.437–0.575), and 16.8% (95% CI, 0.111–0.225), respectively (Figure 4B). In the entire group, the corresponding figures were 23.0 months (95% CI, 20.5–25.5), 12.0 months (95% CI, 11.1–12.9), and 5.0 months (95% CI, 4.4–5.6), respectively, and the 1-year OS rate in the three risk groups was 68.8% (95% CI, 0.651–0.725), 48.5% (95% CI, 0.446–0.524), and 21.8% (95% CI, 0.187–0.249), respectively (Figure 4C). Remarkable OS differences were documented among the three risk groups (all *P* < 0.001).

## Discussion

Considerable heterogeneity affects the implementation of clinical decisions for mCC patients in routine oncological practice, but according to the results of this study, we showed that there is still room to improve the survival outcomes for at least some mCC patients. In this adverse clinical scenario, being able to predict the OS time of patients newly diagnosed with mCC and to provide individualized disease-related risk estimations was achieved in this study. We first constructed a novel nomogram model depended on the results from Cox regression analysis. In this nomogram model, we found that several factors were significantly correlated with OS. It is worth mentioning that local surgery was the second most important prognostic factor in this model. Previously, Zhang et al. also built a nomogram for mCC patients from the 2010–2015 SEER data (13). Surgery was initially demonstrated to be a significant prognostic parameter in univariate analysis (*P* < 0.001, HR = 0.525), but was omitted from the final model due to none significant difference in the multivariate analysis. However, different opinions regarding the role of local surgery in mCC patients were also reported in two other SEER studies. In an earlier study that included 1992–2013 mCC patients, the authors' multivariate Cox regression analysis demonstrated a significantly different OS time between local surgery and non-surgical treatment before (*P* < 0.001, HR = 0.52) and after (*P* < 0.001, HR = 0.49) propensity score matching (PSM). Similar OS benefits were also demonstrated for RT and CT (19). In another paper, Li et al. further investigated the value of multimodal therapy consisting of local surgery combined with RT and CT using the same timeframe (2010–2015 in SEER) data as in Zhang's report (20). Their results demonstrated that local surgery combined with RT and CT tended to significantly prolong OS in mCC patients compared with non-surgical treatment (*P* < 0.001, HR = 0.36). Additionally, advanced T stage was also confirmed to be an independent risk parameter for OS, which was compatible with the results in the current study. Another concern regarding Zhang's model is the pathological type. Squamous cell carcinoma (SCC) has been demonstrated to be the worst pathological type in terms of OS relative to adenocarcinoma or other histological types. Two large-sample, retrospective database studies of cervical carcinoma from the SEER (21, 22) and National Cancer Database (23, 24) revealed that SCC was an independent prognostic parameter favoring OS. Thus, caution should be used when applying Zhang's model in the clinic. Additionally, we divided the situation of patients' distant metastases based on metastatic sites instead of the numbers for the first time. mCC patients involved with only distant LNs had the best OS time compared with those with organ metastasis or even both. As mentioned above, Kim's results demonstrated that for mCC patients with only distant LNs metastasis, combination therapy yielded a significant long-term median OS of over 5 years (6). Another large-sample, retrospective study of 205 cervical carcinoma patients with recurrent diseases demonstrated that mCC patients with distant LNs and pulmonary metastasis had a remarkably good prognosis compared with other metastatic sites with a significantly higher 5-year OS rate of 44.8% (25). Similarly, Yin's results demonstrated that mCC patients initially diagnosed with organ metastasis, liver metastasis had the worst OS time (10.9 vs. 13.1 months; *P* = 0.029, HR = 4.02) and progression-free survival (PFS, 3.7 vs. 7.5 months; *P* = 0.021, HR = 3.77) compared with other metastases. Moreover, CT combined with local treatment (RT or surgery for the primary tumor or metastatic sites) was also observed to significantly correlate with better OS (*P* = 0.012, HR = 0.40) and PFS (*P* = 0.027, HR = 0.42), respectively (26).

Second, a novel risk classification system was constructed depended on the scores obtained from each patient. The median OS time was 23 months in the low-risk patients whereas patients in the high-risk cohort had a mere OS time of no more than half a year. This finding indicated that more aggressive treatment combinations should be considered for high-risk mCC patients to increase the poor survival outcomes and improve patients' quality of life. Not limited to the addition of bevacizumab to CT, current breakthroughs in immunotherapies have also shown promising results in this field (27–29). In the CheckMate 358 trial (30), 24 patients with recurrent or metastatic vaginal, vulvar or cervical tumors were allocated to receive nivolumab monotherapy every 2 weeks. Among them, there were 16 (66.6%) stage IV cervical cancer patients. Follow-up analysis revealed a promising survival results with a median OS time of 21.9 months for cervical carcinoma (95% CI, 15.1–not reached). Earlier in 2017, Keynote-028 phase Ib trial (NCT 02054806) was reported to evaluate the safety and efficiency of pembrolizumab in programmed death-1 (PD-1) positive solid tumors. In a subgroup of patients diagnosed with cervical cancer, 15 (62.5%) patients had distant metastasis. Survival results indicated that the median OS and PFS were 11.0 months (95% CI, 4.0–15.0) and 2.0 months (95% CI, 2.0–3.0), respectively, with manageable toxicities (31). Subsequently, in the Keynote-158 trial, 98 patients diagnosed with advanced cervical cancers were also recruited to receive pembrolizumab monotherapy for up to 2 years. Among them, 92 (93.9%) patients were diagnosed with stage IVB. The median OS time was observed to be 9.4 months (95% CI, 7.7–13.1) and 11.0 months in the total population and in the PD-1 positive subpopulation, respectively (32). Except immunotherapies targeting PD-1, a pilot study investigated the safety and efficiency of HPV-targeted Tumor infiltrating lymphocytes (TIL) in mCC patients. Surprisingly, two of nine mCC patients were documented to have complete regression (CR) of cervical tumors and one additional patient experienced partial response. Additionally, tumor regression was continued to over 22 and 15 months after HPV-TIL therapy for CR patients, respectively (33). Hence, combined with the progress in surgical techniques (34) or RT developments such as stereotactic body RT for curative treatment at metastatic sites (35), it is necessary to consider a multimodal treatment strategy based on each patient's condition to improve the survival outcomes of mCC patients.

It is essential to acknowledge that the present study has some limitations. First, this was a retrospective analysis of data registered in the SEER database. Other drawbacks include the unknown data for variables registered in the database and other unavailable information, such as performance status, HPV infection status, comorbidities, CT regimens, RT dosage and subsequent treatment options for metastatic sites. Therefore, some unavailable clinical factors could not be included and further analyzed in the prognostic model. Furthermore, although the nomogram was constructed using a large sample size and further validated in a subgroup of mCC patients from SEER, the limitations of the nomogram and its power to predict specific clinical endpoints need to be taken into account (36). Finally, this nomogram and risk classification system was built based on data registered only in the U.S.A. Thus, caution is warranted in applying the model to other countries or demographics around the world, and its findings need to be confirmed by well-designed large prospective studies.

## Conclusions

To sum up, we constructed a novel nomogram for calculating the OS time of mCC patients between 2010 and 2016 from the SEER database using five clinicopathological characteristics and three treatment-related parameters. Validation in the external cohort suggested its satisfactory performance. Furthermore, a novel risk classification system was effectively built to stratify mCC patients into three different risk categories. These models could be useful in survival prediction and should be validated in future studies.

## Data Availability Statement

The original contributions presented in the study are included in the article/Supplementary Material, further inquiries can be directed to the corresponding authors.

## Ethics Statement

Ethical review and approval was not required for the study on human participants in accordance with the local legislation and institutional requirements. Written informed consent for participation was not required for this study in accordance with the national legislation and the institutional requirements.

## Author Contributions

WY: conception and design, drafting, final approval, and accountable for aspects. LH and ZZ: provision of study materials, collection and assembly of data, drafting, final approval, and accountable for aspects. TS: conception and design, provision of study materials, collection and assembly of data, drafting, final approval, and accountable for aspects. HX: conception and design, provision of study materials, collection data, drafting, final approval, and accountable for aspects. YJ, JH, and HS: data analysis and interpretation, drafting, final approval, and accountable for aspects. All authors contributed to the article and approved the submitted version.

## Conflict of Interest

The authors declare that the research was conducted in the absence of any commercial or financial relationships that could be construed as a potential conflict of interest.

## Supplementary Material

Supplementary Figure 1 Flow chart of the process of data extraction from the SEER database.

Supplementary Figure 2 Screen capture of the X-tile software for the cutoff point selection.
