# Supplementary figures and images for: A Nomogram-Based Risk Classification System Predicting the Overall Survival of Patients With Newly Diagnosed Stage IVB Cervix Uteri Carcinoma
Source: Front Med (Lausanne). 2021 Jul 15;8:693567. doi: 10.3389/fmed.2021.693567 (PMC8319470; doi:10.3389/fmed.2021.693567)

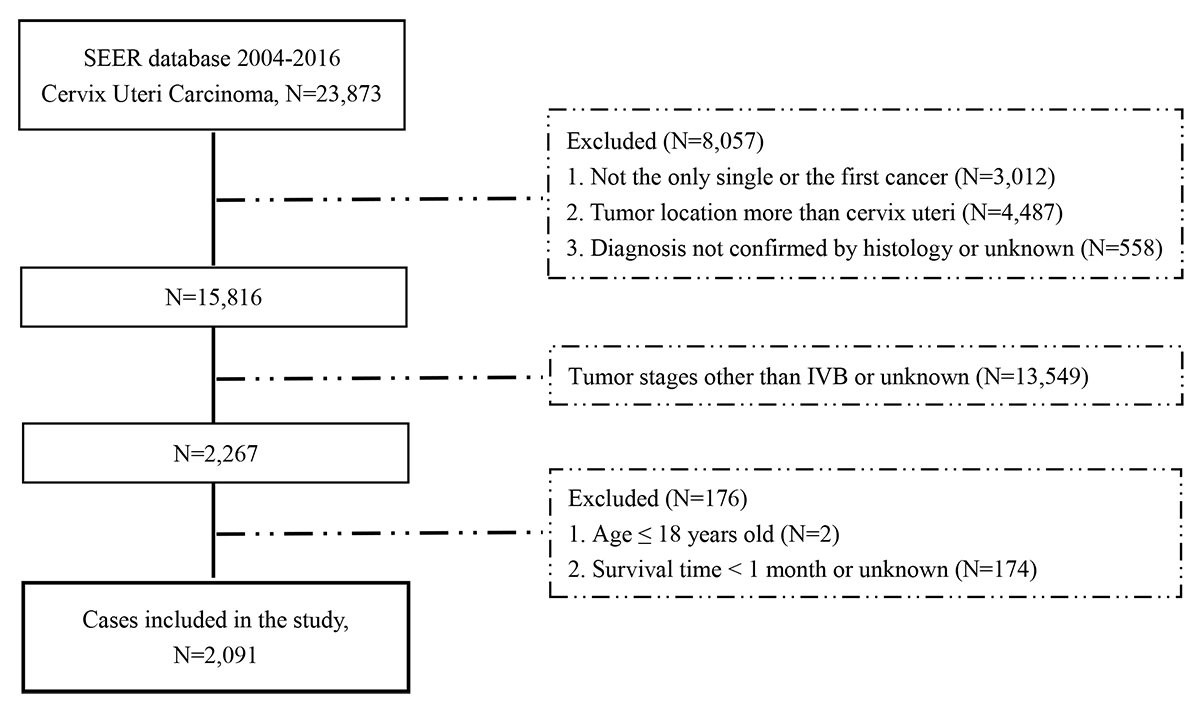

Supplement: Supplementary Figure 1 — Flow chart of the process of data extraction from the SEER database. [file Image_1.TIF]

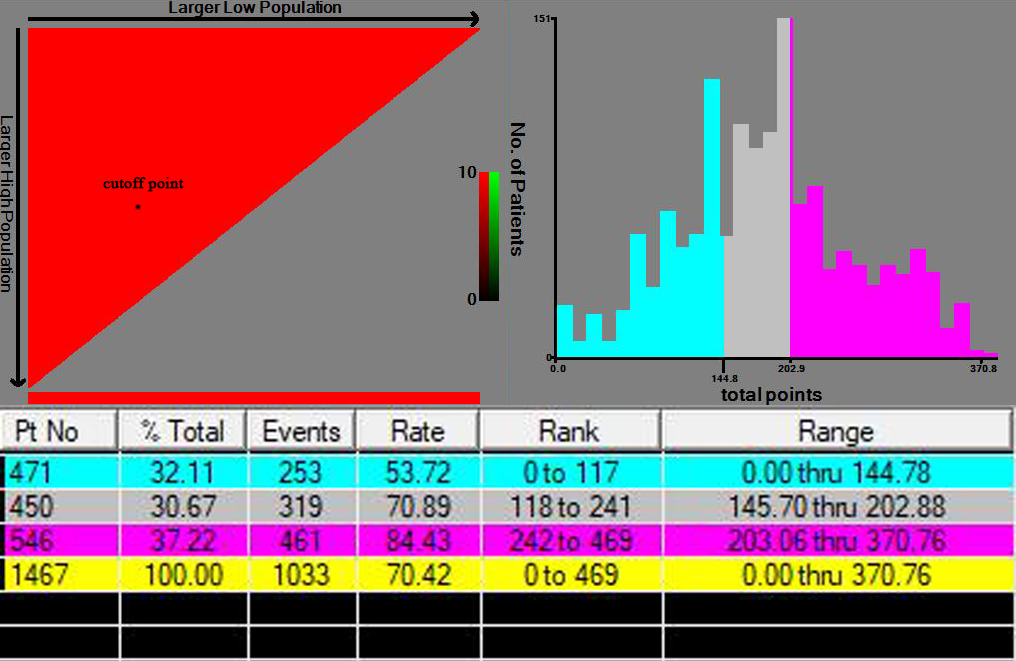

Supplement: Supplementary Figure 2 — Screen capture of the X-tile software for the cutoff point selection. [file Image_2.TIF]
